# Metabolomic Profiling of the Effects of Melittin on Cisplatin Resistant and Cisplatin Sensitive Ovarian Cancer Cells Using Mass Spectrometry and Biolog Microarray Technology

**DOI:** 10.3390/metabo6040035

**Published:** 2016-10-13

**Authors:** Sanad Alonezi, Jonans Tusiimire, Jennifer Wallace, Mark J. Dufton, John A. Parkinson, Louise C. Young, Carol J. Clements, Jin Kyu Park, Jong Woon Jeon, Valerie A. Ferro, David G. Watson

**Affiliations:** 1 Strathclyde Institute of Pharmacy and Biomedical Sciences, University of Strathclyde, Glasgow G4 0RE, UK; alonezi-sanad-mohammed-z@strath.ac.uk (S.A.); jonans.tusiimire@strath.ac.uk (J.T.); louise.c.young@strath.ac.uk (L.C.Y.); c.j.clements@strath.ac.uk (C.J.C.); v.a.ferro@strath.ac.uk (V.A.F.); 2 WestCHEM, Department of Pure and Applied Chemistry, University of Strathclyde, 295 Cathedral Street, Glasgow G1 1XL, UK; jennifer.wallace.101@strath.ac.uk (J.W.); mark.dufton@strath.ac.uk (M.J.D.); john.parkinson@strath.ac.uk (J.A.P.); 3 Beesen Co. Ltd., Bio Venture Town, Yuseong Daero 1662, Dae Jeon 34054, Korea; jkypark@live.co.kr (J.K.P.); confessor@hanmail.net (J.W.J.)

**Keywords:** metabolomics, Melittin, LC-MS, ovarian cancer, A2780 cells, cisplatin resistance

## Abstract

In the present study, liquid chromatography-mass spectrometry (LC-MS) was employed to characterise the metabolic profiles of two human ovarian cancer cell lines A2780 (cisplatin-sensitive) and A2780CR (cisplatin-resistant) in response to their exposure to melittin, a cytotoxic peptide from bee venom. In addition, the metabolomics data were supported by application of Biolog microarray technology to examine the utilisation of carbon sources by the two cell lines. Data extraction with MZmine 2.14 and database searching were applied to provide metabolite lists. Principal component analysis (PCA) gave clear separation between the cisplatin-sensitive and resistant strains and their respective controls. The cisplatin-resistant cells were slightly more sensitive to melittin than the sensitive cells with IC_50_ values of 4.5 and 6.8 μg/mL respectively, although the latter cell line exhibited the greatest metabolic perturbation upon treatment. The changes induced by melittin in the cisplatin-sensitive cells led mostly to reduced levels of amino acids in the proline/glutamine/arginine pathway, as well as to decreased levels of carnitines, polyamines, adenosine triphosphate (ATP) and nicotinamide adenine dinucleotide (NAD+). The effects on energy metabolism were supported by the data from the Biolog assays. The lipid compositions of the two cell lines were quite different with the A2780 cells having higher levels of several ether lipids than the A2780CR cells. Melittin also had some effect on the lipid composition of the cells. Overall, this study suggests that melittin might have some potential as an adjuvant therapy in cancer treatment.

## 1. Introduction

Growth of cancers is associated with various metabolic changes at the cellular level, which can be used as biomarkers for diagnosis, prognosis and evaluation of anticancer therapies [1]. For instance, unlike normal cells, cancer cells are more dependent on aerobic glycolysis, fatty acid synthesis, and glutaminolysis for proliferation [2]. Thus, evaluation of the concentrations of specific metabolites in a cell can provide insights into its metabolic state relative to the physiological norm. The metabolic profile of cancer cells can also provide an understanding of the process of carcinogenesis and the mechanism of chemoresistance leading to development of better diagnostic and therapeutic tools [3].

Worldwide, more than 230,000 women are diagnosed with ovarian cancer each year, and this disease is responsible for an estimated 140,000 deaths per year [4]. Platinum (e.g., cisplatin) and taxane (e.g., paclitaxel)-based chemotherapies are currently the first line treatments for ovarian cancer, but relapse occurs in 70% of patients [3]. Although most ovarian cancers remain sensitive to these therapies, there is growing resistance against them which reduces the time to disease progression following the initial treatment, and minimises their efficacy upon further treatment during relapse [5]. The anticancer activity of platinum arises from its ability to form irreparable intra-strand DNA crosslinks/adducts, which leads to cell apoptosis [6], as well as induction of oxidative and endoplasmic reticulum stress [7,8,9]. On the other hand, platinum resistance in cancer results from various adaptive mechanisms including reduced cellular uptake, increased DNA repair and tolerance [10], and inactivation by glutathione [10,11]. It has been previously reported that platinum-sensitive (A2780) and resistant (C200) ovarian cancer cell lines have distinct metabolic profiles in various interdependent pathways [3].

Melittin, is the main component of honey bee venom and has demonstrated a variety of biological and pharmacological applications [12,13]. It has natural anti-bacterial, anti-viral, and anti-inflammatory properties [12,13]. It has also been shown to have diverse anticancer effects in several different cancer cell lines including those of gastric [14,15], breast [12,16], ovarian [16,17], liver [18,19], prostate [16], cervical [16], and lung [20] origins. The mechanisms by which melittin, an amphipathic haemolytic peptide, exerts its potential anticancer effects include inhibition of cell proliferation [12,17], induction of apoptosis [12,14,15,21], and direct necrosis [14,15]. The mechanism of apoptosis appears to be related to the activation of the caspase-dependent pathway [15,16,21]. On the other hand, necrosis arises from damage to the cell membranes through necrotic cytotoxicity, as has been observed in rat thymocytes, murine skeletal muscle cells, gastrointestinal (GI) tumour cells, erythrocytes, lymphocytes, lymphoblastoid cells, rat primary alveolar cells, and intestinal Caco-2 cells [15,21]. Melittin can also cause cell cycle arrest leading to inhibition of cell proliferation and growth. It can contribute to inhibition of angiogenesis through its ability to suppress epidermal growth factor (EGF)-induced vascular endothelial growth factor (VEGF) secretion and inhibit the creation of new blood vessels by influencing hypoxia-inducible factor (HIF)-1a [22]. Previous studies on ovarian cancer cells have shown that melittin inhibits their growth through induction of apoptosis mediated via inhibition of signal transducer and activator of transcription 3 (STAT3) and activation of Janus kinase 2 (JAK2), both of which are critical during angiogenesis [16]. Melittin can also prevent EGF-induced cell invasion through its inhibition of the PI3K/Akt/mTOR signaling pathway, but this is primarily related to breast cancer cells [12]. In hepatocellular carcinoma, melittin appears to inhibit cell proliferation through its influence on methyl-CpG binding protein 2 (MeCP_2_), which is a critical element in tumour growth and development [19]. As a consequence, melittin induces a delay in G_0_/G_1_ cell cycle progression, which it is able to accomplish without causing apoptosis [19]. Based on these observations, it is apparent that melittin affects cancer cells in a variety of ways that are different from those induced by platinum-based agents. This difference in molecular action could be reflected at cellular level in terms of differences in the metabolite profiles, and would suggest an opportunity for synergy between the two agents and a possible lack of cross-resistance. By determining the metabolite differences between platinum-sensitive and resistant cancer cells after treatment with melittin, it could be possible to understand the latter’s metabolic effects on these cells in relation to their platinum chemosensitivity.

Metabolomics is a growing and powerful technology capable of detecting hundreds to thousands of metabolites in tissues and biofluids [23,24,25,26]. With recent advances in both instrumental and computational metabolomics technologies, it is now possible to gain deeper understanding of the metabolic processes occurring within cancer cells, including how they exploit the process of glycolysis. Cancer cells are known to rely on higher rates of glycolysis, an observation that is known as the “Warburg effect”. With metabolomic profiling, it is possible to relate the “Warburg effect” to the production of amino acids, nucleotides, and lipids necessary for tumour proliferation and vascularisation [27]. Some researchers have suggested that metabolic profiling can be an invaluable tool in the evaluation of drug targets and analysis of malignant phenotypes, including the diagnosis of cancer [28]. For instance, by comparing the metabolite profile of cancer cell lines such as ovarian cancer cells pre- and post-treatment, it is possible to identify relevant metabolic changes that relate to specific treatments, which not only helps in determining the efficacy of the treatment, but is also essential in elucidating its pharmacodynamics and identifying the essential biomarkers involved. Thus, metabolomic analysis of lysates from cell cultures has many potential applications and advantages over conventional methods of analytical biochemistry and cell line testing. It is anticipated that as more robust platforms for metabolomics of cell cultures become available, this will facilitate greater understanding of drug actions both in vitro and in vivo, as well as aiding the rapid incorporation of drug leads into novel therapeutic agents [29].

Phenotype Microarrays (PMs) use a patented redox chemistry, employing cell respiration as a universal reporter. These assays potentially provide a natural fit to support data obtained from metabolomics screens. The redox assay provides for both amplification and precise quantitation of phenotypes. Redox dye mixes contain a water-soluble nontoxic tetrazolium reagent that can be used with virtually any type of animal cell line or primary cell [30]. The dyes used in Biolog (Hayward, CA, USA) assays measure output of nicotinamide adenine dinucleotide reduced form (NADH) production from various catabolic pathways present in the cells being tested. If cell growth is supported by the medium in an assay well, the actively metabolizing cells reduce the tetrazolium dye. Reduction of the dye results in colour formation in the well, and the phenotype is considered “positive.” If metabolism is hampered or growth is poor, then the phenotype is “weakly positive” or “negative,” and little or no colour is formed in the well. This colorimetric redox assay allows examination of the effect of treatment on the metabolic rate produced by different substrates and thus is an excellent technique to combine with examination of metabolic output via metabolomics screens.

The current study sought to examine the metabolic effects of melittin on cisplatin-resistant (A2780CR) and cisplatin-sensitive (A2780) human ovarian cancer cell lines via mass spectrometry-based metabolic profiling in combination with Biolog microarray assays. Each of the cell lines was separately treated with sub-lethal doses of melittin for 24 h before extraction and global metabolite analysis of cell lysates by LC-MS using a high performance liquid chromatography (HPLC) system coupled to an Orbitrap Exactive mass spectrometer using a ZIC-pHILIC column. The resulting data were extracted by MZMine and subsequently analysed by univariate and multivariate approaches with SIMCA-P.

## 2. Results

### 2.1. Melittin Sensitivity of the Ovarian Cancer Cells

**Figure 1:**
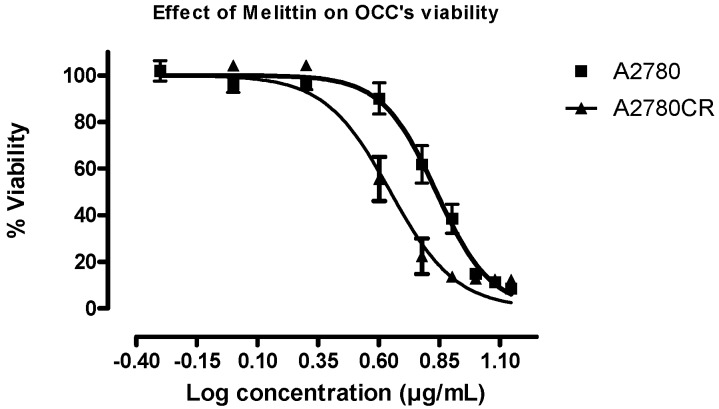
Effect of melittin on the viability of the ovarian cancer cells A2780 and A2780CR. Cell viability was determined following treatment with varying doses of melittin for 24 h (IC_50_ = 6.8 µg/mL A2780; IC_50_ = 4.5 µg/mL A2780CR).

Figure S1 shows the cell viability plots for A2780 and A2780CR cells treated with cisplatin. Melittin exhibited toxicity against both A2780CR and A2780 cells, with IC_50_ values of 4.5 and 6.8 μg/mL, respectively (Figure 1). Thus, the cisplatin-resistant A2780CR cells were more sensitive to melittin than the cisplatin-sensitive A2780 cells and exhibited a response curve suggestive of a dose-response relationship.

### 2.2. Phenotypic MicroArray (PM) Assay of Untreated and Melittin Treated A2780 and A2780CR Cells

**Figure 2:**
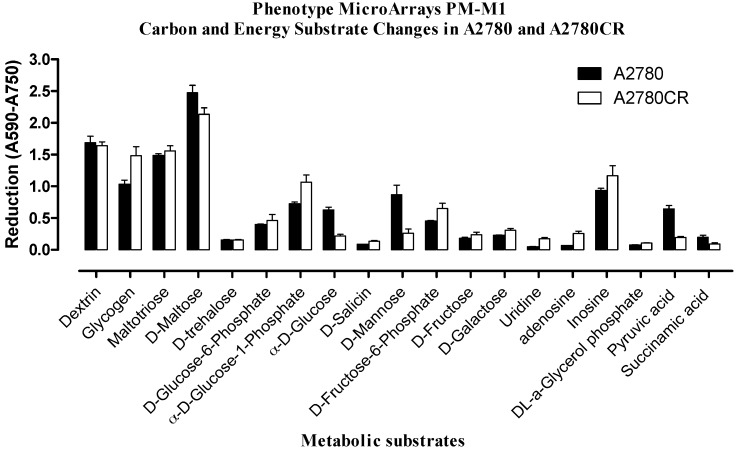
Comparison of substrate metabolism in A2780 and A2780CR. Dye reduction rates calculated following 24 h incubation of cells.

The cells were tested by using standard protocols for metabolic phenotype microarray-mammalian (PM-M) cell assays (Biolog, Hayward, CA, USA). In order to select the proper dye mix, an optimisation experiment was performed in order to determine which of the two Biolog redox dyes (MA or MB) was most appropriate for phenotypic microarray (PM) assay of both A2780 and A2780CR cells. Figure S2 shows the layout of the carbon sources in the PM-M1 microplate. Figure S3 shows the colours which developed in the plates after inoculation with A2780 and A2780CR cells in the presence and absence of melittin. Figure 2 indicates the extent of utilisation of the different carbon sources by the resistant and sensitive cells. A number of the carbon sources were used by both cell lines. However, many of the substrates in the microarray plate do not appear to be useful as carbon sources. There were clear phenotypic differences and the A2780CR cells would appear to have a slightly more active glycolytic pathway as judged by the rate of utilization of glucose and fructose phosphates, although the rates of glucose and pyruvate utilisation were higher in the A2780 cell line. Inosine also appears to be a very favourable carbon source and is used by the A2780CR cells to a greater extent than by the A2780 cells. Neither Krebs cycle intermediates nor short chain fatty acids appear to be useful as carbon sources underlining the dependence of both cell lines on glycolysis, which is generally the case in cancer cell lines [2].

**Figure 3:**
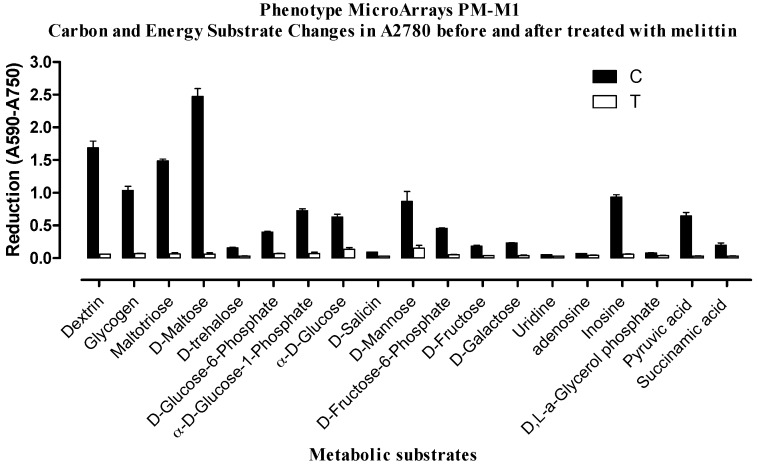
Comparison of substrate metabolism in A2780 cells following melittin exposure. Dye reduction rates calculated following 24 h incubation of cells with melittin at IC_50_ (6.8 µg/mL) concentration. C = untreated controls; T = melittin treated.

**Figure 4:**
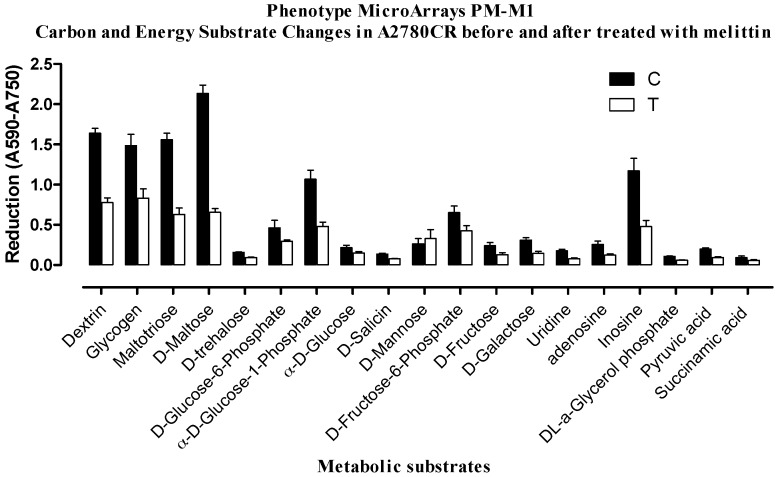
Comparison of substrate metabolism in A2780CR cells following melittin exposure. Dye reduction rates calculated following 24 h incubation of cells with melittin at IC_50_ (4.5 µg/mL) concentration. C = untreated controls; T = melittin treated.

Treatment of the cells with melittin produced a very different effect on the sensitive cells in comparison with the resistant cells. In the resistant cells carbon metabolism was not that strongly affected and the cells continued to produce NADH, but in the sensitive cells there was a huge reduction in carbon metabolism (Figure 3 and Figure 4). This suggests that there may be differences in the mechanisms by which the two cell lines respond to melittin, which could lead to different mechanisms of cell death induced by the treatment.

### 2.3. Effect of Melittin on the Metabolomes of Both Cell Lines

**Figure 5:**
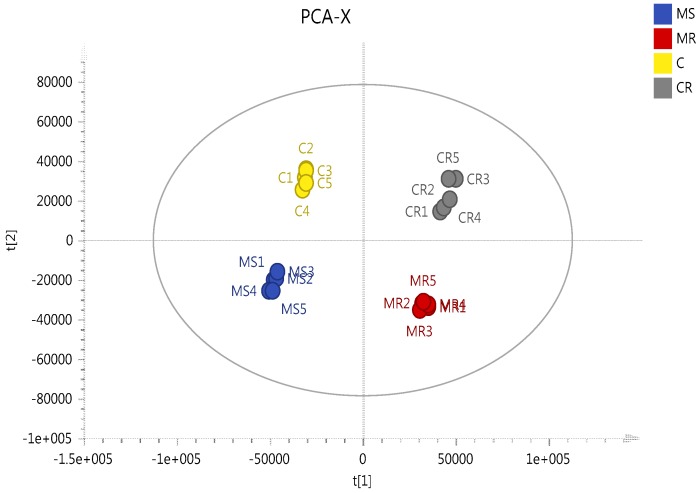
Multivariate data analysis of the ovarian cancer cells A2780 and A2780CR treated with melittin. PCA scores plot generated from PCA using LC-MS normalised data of treated cells and controls. MS circles: A2780-treated cells; C circles: untreated A2780 cells; MR circles: A2780CR -treated cells; CR circles: untreated A2780CR.

In order to gain a better understanding of the mechanism of melittin toxicity in the two cell lines, differences in the levels of metabolites induced by treatment with melittin at concentrations corresponding to IC_50_ with respect to each cell line were assessed. PCA and HCA analyses were used to classify the metabolic phenotypes and identify the differentiating metabolites. A clear separation of melittin-treated A2780 and A2780CR cells, and their respective untreated controls, was achieved indicating unique metabolite profiles for the treated and control cells on a PCA scores plot (Figure 5). The model parameters and validation of the plot suggested a good model (2 components, *R*^2^*X* = 0.814; *Q*^2^ = 0.526). The HCA groupings of the metabolomics data showed distinct separation between the cell lines themselves as well as between the control and treated samples of each cell line.

Table 1 shows the metabolic differences between the sensitive and resistant cell lines. The low levels of ATP in the A2780 cells reflected the Biolog data suggesting that these cells have reduced rates of glycolysis in comparison with the resistant cells. Several metabolites in the TCA cycle differed between the sensitive and resistant cells including citrate, 2-oxoglutarate, and malate. None of the TCA intermediates supplied in the Biolog array were utilised by the cell lines as carbon sources. However, pyruvate was used as a carbon source and presumably enters the Krebs cycle. Treatment with melittin further reduced the levels of ATP in the A2780 cells whereas melittin had little effect on the ATP levels in the A2780CR cells. There were marked differences in levels of some carnitines between the sensitive and resistant cells with the sensitive cells having much higher levels of butyryl carnitine. However, there was no evidence from the Biolog data that short chain fatty acids were utilised as carbon sources.

The most marked differences between the sensitive and resistant cells were in the polyamine pathway where 14 metabolites in the pathway were altered in the sensitive cells in comparison with the resistant cells. The polyamines spermidine, putrescine and *N*-acetylputrescine were markedly higher in the sensitive cells and, correspondingly, many of their precursors, especially arginine, were down regulated. Melittin treatment decreased the levels of polyamines in the sensitive cells and the levels of arginine present in the cells were reduced almost to zero.

**Figure 6:**
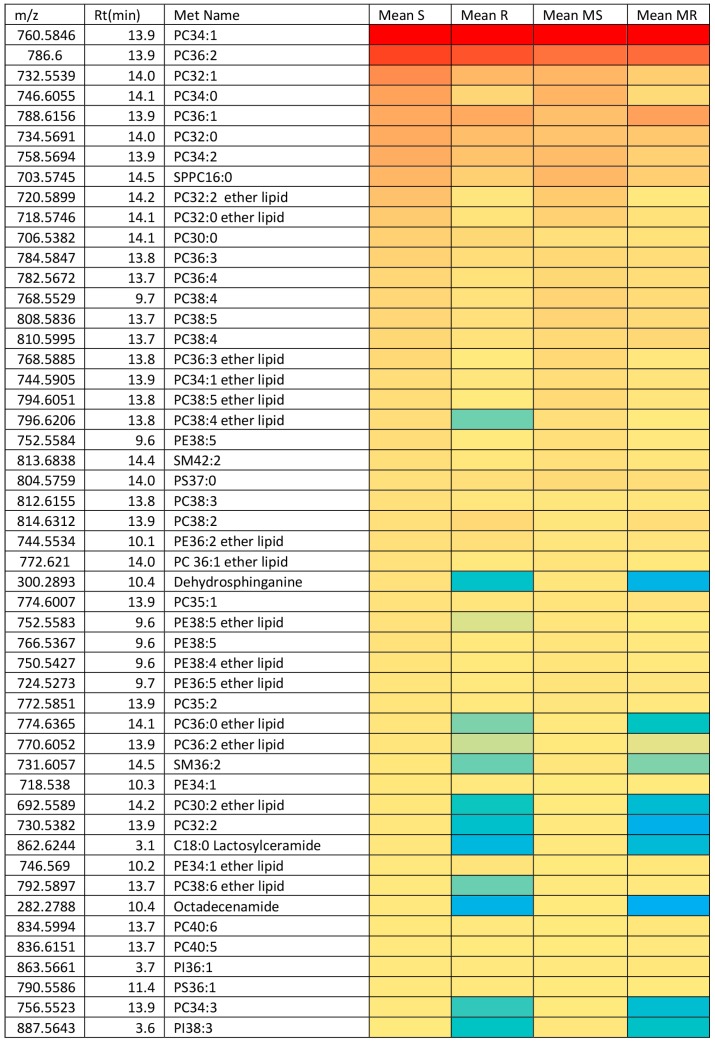
Heat Map showing the relative abundance of lipids in A2780 (S), A2780CR (R) and melittin treated (MS & MR) cells. Red = 5 × 10^7^, Yellow = 8% of highest value, Blue = 0%.

There were important differences in lipid composition between the two cell lines before and after treatment with melittin. Figure 6 shows a heat map of the top 50 lipids by intensity extracted from the A2780 cells in comparison with A2780CR cells. The top 10 most abundant lipids are similar between the two cell lines and make up a large proportion of the lipids extracted. However, it is clear that the A2780 cell line generally contains more lipids than the A2780CR cell line. The really marked differences between the two cell lines are in several sphingolipids such as dehydrosphinganine and lactosylceramide, and in some ether lipids such as PC38:4 and PC38:6, all of which are lower in the resistant cells. The differences in lipid composition of the two cell lines suggest that either remodelling of the cell membrane might have occurred in order for the A2780CR cells to become resistant, or there is decreased biosynthesis and/or increased utilisation of lipids in cisplatin resistant cells as has been suggested by others [3]. Melittin appears to have some effect on lipid composition in the A2780 cells with the levels of the abundant lipid PC34:1 decreasing, but the effect on this lipid in the A2780CR cells is less marked. Overall there are many changes in lipid abundance in response to melittin but they are generally quite small and restricted to the less abundant lipids (Table S1). The decrease in the lipids induced by treatment with melittin is less in the case of the A2780CR cells suggesting less membrane damage in the case of these cells.

### 2.4. Assessment of Necrotic and Apoptotic Cell Death in A2780 and A2780CR Cells

LDH (lactate dehydrogenase) release in the medium is an enzymatic indicator that illustrates the breakdown of membrane integrity, apoptosis, or necrosis of a cell. A2780 and A2780CR cancer cells were incubated with increasing concentrations of melittin for 24 h and intracellular LDH release increased as a result of the breakdown of the cell or plasma membrane. The results suggest that compared to control cells (untreated cells), melittin produced an increase in LDH leakage when incubated with ovarian cancer cells at levels ≥6 μg/mL (Figure S4); the amount of LDH released appeared to be concentration-dependent in A2780, but not in A2780CR, cells. However, in comparison to staurosoporine, melittin did not induce high levels of caspase activity particularly at 6 h (Figure S5). This suggests that the mechanism of cell death promoted by melittin was via necrosis rather than apoptosis.

## 3. Discussion

In this study, untargeted metabolomics was performed in order to determine the effects of melittin on the metabolic output of A2780 and A2780CR ovarian cancer cell lines. The altered metabolites in both cells encompassed several pathways including those of lipid, amino acid, energy, and carbohydrate metabolism. In a previous study it was found that docetaxel, the chemotherapeutic agent used for treatment of ovarian cancer, caused significant metabolic changes in amino acid and carbohydrate metabolism in ovarian cancer cells (OVCAR-3) [31]. In addition, recent metabolomics based studies in ovarian cancer cells have demonstrated that gossypol decreases cellular levels of glutathione (GSH), aspartic acid, and flavin adenine dinucleotide (FAD) [32]. Thus, the results obtained with melittin in this study add to the growing body of evidence regarding the utility of metabolomics as a tool for evaluating metabolic alterations in cancer cells induced by various agents. However, while there have been some previous metabolomics studies on the comparison between platinum-sensitive and resistant ovarian cancer cell lines [3], and between effects of nicotinamide phosphoribosyltransferase on ovarian and colorectal cancers [33], this is the first metabolomics-based study to evaluate the effects of melittin on human ovarian cancer cell lines as a potential anticancer therapeutic agent.

The overall impression is that the cisplatin-sensitive cells exhibit a much stronger metabolic response to melittin treatment than the resistant cells, possibly indicating a greater capacity of the former cell line to neutralise the effects of melittin given its higher IC_50_ value against this cell line. In particular, the levels of several amino acids including proline, pyrroline-3-hydroxy-5-carboxylate, glutamate, glutamate-5-semialdehyde, *N*-acetyl-*l*-glutamate, and arginine were all markedly decreased in A2780 cells following melittin exposure. In a previous study by Poisson et al. (2015) to compare the metabolic profiles of untreated cisplatin-resistant and sensitive cell lines [3], arginine was found to be significantly higher in the latter. In contrast, our study shows that arginine was higher in the resistant compared to sensitive cell lines both pre- and post-treatment with melittin. However, it should be noted that the Poisson et al. study [3] compared the sensitive A2780 cells with C200, a different cisplatin resistant cell line from the one used in the current study. Thus, our findings suggest that the A2780CR cell line contains more arginine than both the cisplatin sensitive A2780 and cisplatin-resistant C200 cell lines and, unlike in A2780 cells where it is lowered, the arginine level in A2780CR cells is unperturbed by treatment with melittin. In correspondence to lower levels of intermediates in the arginine pathway, ornithine, putrescine, *N*-acetylputrescine and spermidine were all upregulated suggesting that the levels of arginine and its precursors are lower in the A2780 cells since they are being directed towards polyamine biosynthesis. The lower level of methionine in these cells correlates with an increased requirement for it in the biosynthesis of spermidine. High levels of polyamines have been linked to high rates of cell proliferation. Treatment of the sensitive cells with melittin results in an almost complete depletion of arginine within the cells and further lowering of arginine precursors. The lack of arginine as a precursor appears to result in a fall in the level of ornithine and polyamines within the cells although the levels still remain higher than those in the resistant cells and methionine is higher after treatment suggesting that the requirement for it in spermidine biosynthesis is reduced because of the depletion of spermidine. Polyamine metabolism in the resistant cells remains unaffected by melittin. Polyamines are known to act to stabilise membranes through interaction with phospholipid head groups [34]. It has been speculated that polyamines stabilise membrane flow, which involves fusion between the plasma membrane and Golgi derived vesicles [35,36]. The resistant cells contain higher levels of arginine and lower levels of polyamines suggesting a slower rate of biosynthesis of polyamines from arginine in these cells. Treatment of the resistant cells with melittin does not affect the levels of either the polyamines or arginine to any great extent. If the hypothesis regarding the role of polyamines in stabilising membrane flow is true, then it is possible that the lower levels of polyamines might result in reduced capability in these cells to repair membrane damage caused by melittin. In addition there is a link between polyamine depletion and the inhibition of apoptotic cell death [37]. This further underlines possible differences in the mechanism of cell death between these two cell lines.

The A2780 cells have lower levels of ATP both before and after melittin treatment in comparison with the A2780CR cells. The Biolog data also indicates lower levels of glycolysis in the A2780 cells in comparison with the A2780CR cells. Since ATP generation in cancer cells is primarily from glycolysis as opposed to oxidative phosphorylation, even under normoxic conditions [38], the observed effect implies that glycolysis may be a potential target for melittin as an anticancer agent. The strong dependence of cancer cells on glycolysis could be the basis for melittin’s selective toxicity against them [13,14,15,16,17,18,19]. On the other hand, levels of ATP in A2780CR cells were higher than in A2780 at the outset and were not greatly affected by melittin treatment. The Biolog data also suggested a smaller effect of melittin on ATP production in A2780CR cells since the production of NADH by the cells was much less affected by melittin treatment than in the case of the A2780 cells. ATP levels have been linked to the capability of cells to undergo apoptotic as opposed to necrotic cell death and this suggests that the A2780CR cells may be undergoing apoptotic cell death in response to melittin whereas the A2780 cells may be undergoing necrotic cell death. However, the effect of melittin on caspase levels does not support this. In our study, we found that melittin inhibited glycolysis in A2780 cells by reducing the level of NAD+, but this biomarker was increased in A2780CR cells. Cancer cells require increased NAD+ biosynthesis to support anabolic metabolism, to sustain signalling processes including sirtuin activity and ADP-ribosylation, and to maintain a redox balance. Accordingly, inhibitors of nicotinamide phosphoribosyltransferase (NamPT), the enzyme that catalyses the rate-limiting step in NAD+ biosynthesis, have been shown to possess moderate anti-tumour activity in monotherapy both in vitro and in vivo [39]. The peptide toxin ricin was found to promote apoptosis by decreasing both ATP and NAD levels in U937 cells [40] although it was proposed that necrotic mechanisms might also be operating.

Levels of choline, methionine, phenylalanine, valine and threonine observed were raised in both cell lines when treated with melittin and they were significantly higher in A2780 cells. These findings resemble those from a previous study in which the levels of phenylalanine and methionine were elevated in A2780 and HCT-116 (colorectal cancer) cell lines following treatment with FK866, a small molecule inhibitor of NamPT [41]. However, the levels of metabolic intermediates of the TCA cycle, such as citrate, 2-oxoglutarate, and malate were decreased in A2780 cells by melittin, but they were increased in A2780CR. Some recent studies have demonstrated higher levels of TCA cycle intermediates (including succinate, fumarate, and malate) observed in tissue samples from ovarian carcinoma without treatment [42,43,44] and our study shows as well that there are significant differences in citrate and malate levels in the untreated cells (negative controls) in which malate is decreased and citrate is increased in A2780 relative to A2780CR cells respectively. However, the previously mentioned study by Poisson et al. did not find significant differences in TCA cycle metabolites between untreated platinum-sensitive (A2780) and resistant (C200) cells [3], suggesting that the A2780CR cell line has relatively specific distinctions in its metabolome. Some acyl carnitines were also found to respond to melittin treatment in A2780, but not in A2780CR cells. The dose-dependent decreases in *L*-carnitine, acetylcarnitine and butanoylcarnitine levels in A2780 cells after melittin exposure might be explained based on cell-specific alteration of metabolic pathways. Carnitine serves an important role in the regulation of energy production from fatty acids and glucose at the cellular level. It is involved in the transport of long-chain fatty acids across the inner mitochondrial membrane, as well as facilitating chain-shorted acyl group transportation from the peroxisomes, where they are produced, to the mitochondria for further energy metabolism [45].

There are some significant differences in the lipid composition between A2780 cells and A2780CR cells. Several sphingomyelin (SM) lipids are higher in the A2780 cells in comparison to the A2780CR cells. This differs from previous reports of increased ceramide lipids, particularly glucosylceramides and galactosylceramides, in multidrug resistant ovarian cancer cells [46] and breast cancer cells [47]. However, in these previous studies ceramides or glycosylceramides were measured rather than sphingomyelin lipids. In common with Veldman et al. [46], we have observed lower levels of lactosylceramide in the resistant cells. It has also been previously reported that ether lipids are elevated in vinblastine-resistant human leukaemic lymphoblasts compared with sensitive cells [48], although in the current case many ether lipids are elevated in the cisplatin-sensitive cells and lowered in the resistant ones. Ether lipids increase membrane impermeability due to their ability to form hydrogen bonds with cholesterol and their resistance to hydrolysis by phospholipases [48].

The presence of lower levels of many lipids in the resistant cells, particularly those lipids whose function involves promoting membrane stability, suggests that cisplatin resistance in this cell line is unconventional. Cisplatin, a polar drug, does not cross cell membranes by passive diffusion but via action of organic cation transporters of which several have been identified [49]. Thus, it is possible that cisplatin resistance could be mediated through mechanisms other than augmentation of membrane lipids. This possibility is supported by two of our findings which suggest that melittin, an agent known to destabilise cell membranes, was more active on the cisplatin resistant compared to cisplatin sensitive cells, and that polyamines were higher in the sensitive cells. Both observations further illustrate the fact that the A2780 cell line had a more stable cell membrane. Why membrane stability of the A2780 cells would make them more vulnerable to cisplatin still remains unclear.

Following treatment with melittin, lipids were significantly altered in both A2780 and A2780CR cells although the fold changes are quite small. The observed effect was much more marked in the cisplatin-sensitive cells, where there was a larger number of lipids significantly decreased, suggesting that the latter undergo much more extensive membrane re-modelling in response to melittin in comparison with the resistant cells.

## 4. Materials and Methods

### 4.1. Cell Lines and Cultures

The cisplatin-sensitive (A2780) and resistant (A2780CR) human ovarian carcinoma cells were obtained from ECACC (Porton Down, Salisbury, UK) and maintained at 75 × 10^4^ cells/mL in RPMI 1640 medium (Lonza, Verviers, Belgium) supplemented with 1% (*v*/*v*) *L*-glutamine (Invitrogen, Paisley, UK), 100 IU/mL/100 µg/mL penicillin/streptomycin (Invitrogen, Paisley, UK), and 10% (*v*/*v*) foetal bovine serum (FBS) (Life Technologies, Carlsbad, CA, USA). In addition, the cultures for the A2780CR cells contained 1 µM cisplatinum (Tocris Bioscience, Bristol, UK) in the first three passages. Sub-confluent cultures were split by trypsinisation every 4–5 days and maintained at 37 °C in a humidified atmosphere saturated with 5% CO_2_.

### 4.2. Cell Viability Assay against Melittin

Melittin was purified from bee venom (supplied by Beesen Co. Ltd., Dae Jeon, Korea) by reversed phase liquid chromatography [50] and reconstituted in sterile water to form a stock solution of 1 mg/mL before storage at −20 °C until required for analysis. Cell viability was assessed by an Alamar^®^ Blue (AB) cell viability reagent (Thermo Fisher Scientific, Loughborough, UK). Both A2780 and A2780CR cells were seeded at 1 × 10^4^ cells/well in 96-well plates (Corning^®^, Sigma-Aldrich, Dorset, UK) and incubated at 37 °C and 5% CO_2_ in a humidified atmosphere for 24 h. After this incubation period, the cells were treated with various concentrations of melittin ranging from 0.5 to 14 µg/mL in 100 μL of medium, and re-incubated at 37 °C and 5% CO_2_ for a further 24 h. Triton X at 1% (*v*/*v*) and cell culture media were used as positive and negative controls, respectively. After this, AB was added at a final concentration of 10% (*v*/*v*) and the resultant mixture was incubated for a further 4 h at 37 °C and 5% CO_2_. Then, the plates were read at an excitation wavelength of 560 nm and the emission at 590 nm was recorded on a SpectraMax M3 microplate reader (Molecular Devices, Sunnyvale, CA, USA). Background-corrected fluorescence readings were converted to cell viability data for each test well by expressing them as percentages relative to the mean negative control value.

### 4.3. Determination of IC_50_

GraphPad Prism for Windows (version 5.00, GraphPad Software, San Diego, CA, USA) was employed to produce dose-response curves by performing nonlinear regression analysis of the cell viability data. The mean inhibitory concentration (IC_50_) values were calculated from at least three measurements of independent experiments (*n* = 3).

### 4.4. Determination of Effect of Melittin on Cell Metabolomes

The A2780 and A2780CR cell lines were separately treated with melittin at concentrations of 6.8 and 4.5 μg/mL respectively for 24 h (*n* = 5). The cells were seeded at 75 × 10^4^ cells/mL in T-25 cell culture flasks and incubated for 1 doubling time (48 h) before treatment with the melittin and incubation for an additional 24 h. After the treatment, the medium was removed and the cells were washed twice with 3 mL of phosphate-buffered saline (PBS) at 37 °C before lysis. Cell lysates were prepared by extraction with ice cold methanol:acetonitrile:water (50:30:20) (1 mL per 2 × 10^6^ cells). Lipids were extracted with isopropanol (4 °C) (Sigma-Aldrich, Dorset, UK). The cells were scraped and cell lysates mixed on a Thermo mixer at 1440 rotations per minute (r.p.m.) for 12 min at 4 °C, before being centrifuged at 13,500 r.p.m. for 15 min at 0 °C. The supernatants were collected and transferred into HPLC vials for LC-MS analysis. During the analysis, the temperature of the autosampler was maintained at 4 °C. Mixtures of authentic standard metabolites (Sigma-Aldrich, Dorset, UK), prepared as previously described [51], and the pooled quality control (QC) sample, were injected in each analysis run in order to facilitate identification and to evaluate the stability and reproducibility of the analytical method, respectively. The pooled QC sample was obtained by taking equal aliquots from all the samples and placing them into the same HPLC vial.

### 4.5. Optimisation of Phenotype Microarray Experiment Parameters

(1) A2780 and A2780CR cells were cultured in a 75 cm^2^ culture flask containing 10 ml RPMI-1640 medium lacking phenol red but containing 5% (*v*/*v*) FBS, *L*-glutamine and Pen/Strep (Gibco™ by Life Technologies, Paisley, UK).
(2) The medium was removed from the culture flask and saved in a 15 mL sterile conical tube. The remaining medium was aspirated and discarded from the culture flask. The adherent cells were washed twice with 10 mL of Dulbecco’s Phosphate-Buffered Saline (D-PBS) (Gibco, Paisley, UK) and any remaining D-PBS was aspirated and discarded.
(3) The cells were then detached by adding 2 mL of 0.25 % (*v*/*v*) Trypsin-EDTA (Gibco, Paisley, UK) and incubated at 37 °C for 3 min.
(4) Then, 3 mL of culture medium was taken from the 15 mL conical tube was added to quench the detachment reaction and the cell suspension mixed by gently pipetting up and down several times to disperse the cells.
(5) The cells were harvested by transferring the cell suspension to the 15 mL conical tube containing the culture medium and centrifuged at 350× *g* for 5 min. After centrifugation, the medium was aspirated and 10 mL of D-PBS was added. After that, the cell pellet was suspended in the D-PBS by pipetting up and down several times, then centrifuged again at 350× *g* for 5 min.
(6) After the second centrifugation, the medium was aspirated and 10 mL of pre-warmed MC-0 was added. The cell pellet in the MC-0 Assay Medium was suspended by pipetting up and down several times. The MC-0 medium was composed of IF-M1 (Technopath Distribution, Tipperary, Ireland) medium supplemented with 5.3% (*v*/*v*) dialysed foetal bovine serum (dFBS) (Gibco™ by Life Technologies, Paisley, UK), 1.1% of 100× Pen/Strep solution (Gibco™ by Life Technologies, Paisley, UK), and 0.16% (*v*/*v*) of 200 mM glutamine (final concentration 0.3 mM).
(7) The cell number was determined and cell viability was assessed by trypan blue dye exclusion (Sigma-Aldrich, Dorset, UK).
(8) The cells were suspended in enough MC-0 Assay Medium to fill the selected number of PM panels and to achieve a density of 4 × 10^5^ cells/mL.
(9) After that, 50 μL/well of the cell suspension was added on two sets of PM-M1 plates (Technopath Distribution, Tipperary, Ireland) so that each well had 20,000 cells. The first one was used as the control set, where untreated A2780 and A2780CR were cultured. A2780 and A2780CR seeded in the second set of plates were exposed to melittin. Both sets of PMs containing A2780 and A2780CR were first incubated for 24 h to allow cells to catabolise all nutrients in medium MC-0. The treated cells set was subsequently inoculated with 25 μL of Melittin/well of three PM-M1 plates at IC_50_ concentration, while 25 μL of MC-0 medium was added to each well in the control set of three PM-M1 plates.
(10) Then, the PM plates were incubated at 37 °C in a humidified atmosphere with 95% Air-5% CO_2_ for 18 h, after which the Biolog Redox Dye Mix MA was added to all wells (15 µL/well to the plate). The plate was sealed with tape to prevent off-gassing of CO_2_.
(11) The plates were incubated for an additional 6 h with Biolog Redox Dye Mix MA (Technopath Distribution, Tipperary, Ireland).
(12) Tetrazolium reduction was determined with a microplate reader (SpectraMax M3, Molecular Devices, Sunnyvale, CA, USA). The endpoint read was performed at 590 nm with subtraction of a 750 nm reference reading (A590-750) which corrects for any background light scattering.

### 4.6. LC-MS Conditions

Liquid chromatographic separation was carried out on an Accela HPLC system interfaced to an Exactive Orbitrap mass spectrometer (Thermo Fisher Scientific, Bremen, Germany) using a hydrophilic interaction liquid chromatography (HILIC) column (ZICp-HILIC, 150 × 4.6 mm, 5 µm particle size) supplied by Hichrom Ltd. (Reading, UK). Since chromatographic separation of polyamines is poor on a ZIC-pHILIC column [51], a ZIC-HILIC column (150 × 4.6 mm, 5 µm particle size), supplied by Hichrom Ltd. (Reading, UK) was employed for the determination of putrescine and spermidine in the samples. The mobile phase for ZIC-pHILIC consisted of 20 mM ammonium carbonate (Sigma-Aldrich, Dorset, UK) in water purified by Direct-Q 3 Ultrapure water purification system (Millipore, Watford, UK) at pH 9.2 (solvent A) and acetonitrile (Sigma-Aldrich, Dorset, UK) (solvent B) at a flow rate of 0.3 mL/min. The elution gradient was an A:B ratio of 20:80 at 0 min, 80:20 at 30 min, 92:8 at 35 min and finally 20:80 at 45 min as described previously [52]. In the case of the ZIC-HILIC column, the mobile phase was 0.1% (*v*/*v*) formic acid in water (A) and 0.1% (*v*/*v*) formic acid in acetonitrile (B) with a gradient of A:B 50:50 at 0 min, 95:5 from 20–30 min, and 50:50 from 31–36 min. The nitrogen sheath and auxiliary gas flow rates were maintained at 50 and 17 mL/min. The electrospray ionisation (ESI) interface was operated in a positive/negative dual polarity mode. The spray voltage was 4.5 kV for positive mode and 4.0 kV for negative mode, while the ion transfer capillary temperature was 275 °C. Full scan data were obtained in the mass-to-charge ratio (*m*/*z*) range of 75 to 1200 for both ionisation modes with settings of AGC target and resolution as Balanced (1E6) and High (50,000) respectively. Mass calibration was performed for both positive and negative ESI polarities before the analysis using the standard Thermo Calmix solution (Thermo Fisher Scientific, Bremen, Germany) with additional coverage of the lower mass range with signals at *m*/*z* 83.0604 (2 × ACN + H) for the positive and *m*/*z* 91.0037 (2 × HCOO^−^) for the negative modes respectively. The resulting data were recorded using the XCalibur 2.1.0 software package (Thermo Fisher Scientific, Bremen, Germany). Analysis of lipids was carried out on an ACE silica gel column (150 × 4.6 mm, 3 μm, Hichrom, Reading, UK) as described previously [52].

### 4.7. Data Extraction and Analysis

Data extraction for each of the samples was carried out by MZmine-2.10 software [53,54]. The extracted ions, with their corresponding *m*/*z* values and retention times, were pasted into an Excel macro of the most common metabolites prepared in–house to facilitate identification, and a library search was also carried out against accurate mass data of the metabolites in the Human Metabolome, KEGG, and Metlin databases. The lists of the metabolites obtained from these searches were then carefully evaluated manually by considering the quality of their peaks and their retention time match with the standard metabolite mixtures run in the same sequence. All metabolites were within 3 ppm of their exact masses. Statistical analyses were performed using both univariate and multivariate approaches. The p-values from univariate analyses were adjusted using the Bonferroni correction and differences in the levels (or peak areas) of the metabolites between treated and control cells were considered significant at *p* < 0.05. SIMCA-P software version 14.0 (Umetrics, Crewe, UK) was used for unsupervised multivariate analysis of the metabolite data with Pareto scaling prior to principal component (PCA) and hierarchical clustering (HCA) analyses.

### 4.8. LDH Assay

The cytotoxicity of the melittin was determined by the lactate dehydrogenase (LDH) release assay on A2780 and A2780CR cells. LDH release into the medium is due to the loss of membrane integrity either due to apoptosis or necrosis. Briefly, A2780 and A2780CR cells were seeded at 1 × 10^4^ cells/well in 96-well plates and incubated at 37 °C and 5% CO_2_ in a humidified atmosphere for 24 h. The cells were treated with different concentrations of the melittin for 24 h. Then, the supernatant (50 μL) of the treated cells was transferred into 96-well flat-bottomed plates, and 50 μL of the LDH reaction mix (Lactate Dehydrogenase Activity Assay Kit, MAK066, Sigma-Aldrich, Dorset, UK) was added for 30 min. Finally, the intensity of orange colour in the samples indicating the LDH activity was measured at 490 nm. LDH release increased in a dose-dependent manner in melittin treated A2780 and A2780CR cells compared with untreated cells. The values are represented as the means ± SD of three separated experiments.

### 4.9. Caspase Activity Assay

Fluorometric assays of caspase activity were carried out by using the substrate Ac-DEVD-AMC (BD Pharmingen, San Diego, CA, USA) for caspase-3. Both A2780 and A2780CR cells were seeded at 1 × 10^4^ cells/well in costar 96-well black plates and incubated at 37 °C and 5% CO_2_ in a humidified atmosphere for 24 h. Then, the cells were treated for 6 and 24 h with different concentrations of melittin to measure caspase-3 activity. Staurosporine (Sigma-Aldrich, Dorset, UK) was used to induce apoptosis at a concentration of 10 μM. The control cells were treated with media alone. The caspase-3 assay buffer was prepared as described previously [55]. The caspase-3 assay buffer (3×) was added to each well and incubated at 37 °C in 5% CO_2_ for 1 h. Fluorescence was measured at 360 nm (excitation) and 460 nm (emission) using a Spectramax M3 microplate reader. The average fluorescence values of the background were subtracted from the fluorescence values of experimental wells. Statistical analysis was done using one-way ANOVA followed by Bonferroni’s Multiple Comparison test.

## 5. Conclusions

In conclusion, this study shows that the cisplatin sensitive A2780 cells contain relatively higher levels of ether lipids and polyamines, which might result in increased membrane stability and repair and thus resistance to the lytic action of melittin in comparison with the cisplatin resistant A2780CR cells. After exposure to melittin, the levels of most of the significantly altered metabolites, particularly amino acids and TCA cycle intermediates, were lower in A2780 compared to A2780CR cells, suggesting different metabolic responses in the two cell lines. The large increases in choline and glycerophosphocholine in A2780 cells may be related to increased de novo lipid synthesis and re-direction of cellular metabolism. Thus, analysis of the full lipidome could offer a more valuable insight. Given that melittin interacts with cell membranes, the observed effects might suggest that the membranes are less adaptable in the cisplatin resistant cells compared to the sensitive ones. Over all, this study shows that a LC-MS based metabolomics approach for the assessment of drug effects in vitro provides a powerful tool for obtaining insights into the mechanism of action of potential therapeutic agents, while offering the possibility to identify key metabolite markers for in vivo monitoring of tumour responsiveness to standard chemotherapy. Melittin might serve as a valuable adjuvant in cancer chemotherapy for overcoming chemoresistance.

## Figures and Tables

**Table 1:** Statistically differentiating metabolites in melittin treated and untreated A2780 and A2780CR cells.

*m*/*z*	RT	Metabolite Name	S/R		MS/MR		MR/R		MS/S	
			*p*-Value	Ratio	*p*-Value	Ratio	*p*-Value	Ratio	*p*-Value	Ratio
**Proline/glutamate/arginine/polyamine metabolism**										
116.071	12.8	* Proline	<0.01	0.837	<0.001	0.409	ns	1.056	<0.001	0.516
128.035	10.3	1-Pyrroline-3-hydroxy-5-carboxylate	<0.05	0.839	<0.001	0.364	ns	1.114	<0.001	0.453
130.051	14.4	Glutamate-5-semialdehyde	ns	0.944	<0.001	0.607	ns	1.065	<0.001	0.658
131.083	11.4	* Ornithine	<0.001	4.774	<0.001	3.573	ns	1.001	<0.001	0.749
132.030	15.2	* Aspartate	ns	1.091	<0.001	1.229	ns	1.055	<0.01	1.159
146.046	10.8	Glutamate	<0.001	0.608	<0.001	0.113	ns	0.999	<0.001	0.187
147.076	14.9	* Glutamine	<0.001	0.299	<0.001	0.503	<0.001	1.204	<0.001	1.906
173.104	24.6	* Arginine	<0.001	0.155	<0.001	0.004	ns	1.023	<0.05	0.026
188.057	14.4	* *N*-Acetyl-l-glutamate	<0.001	0.637	<0.001	0.146	<0.001	1.348	<0.001	0.313
89.107	15.4	** Putrescine	<0.001	2.339	<0.001	1.490	ns	1.051	<0.001	0.670
131.118	8.24	*N*-Acetylputrescine	<0.001	5.175	<0.001	1.603	ns	1.021	<0.001	0.316
146.165	26.2	** Spermidine	<0.001	2.354	ns	1.150	ns	1.127	<0.001	0.550
150.058	11.4	* Methionine	<0.001	0.422	<0.001	0.669	<0.01	1.127	<0.001	1.594
298.096	6.42	* 5′-Methylthioadenosine	0.003	1.92	ns	0.830	ns	0.839	<0.001	0.361
**TCA cycle/glycolysis**										
133.014	16.4	* Malate	<0.001	0.647	<0.001	0.238	<0.001	1.129	<0.001	0.418
145.014	15.9	* 2-Oxoglutarate	ns	0.972	<0.001	0.173	<0.001	1.217	<0.001	0.221
191.020	18.4	* Citrate	<0.001	2.207	<0.001	0.534	ns	1.098	<0.001	0.265
508.003	16.6	* ATP	<0.001	0.267	<0.001	0.118	ns	0.963	<0.001	0.415
**Carnitine metabolism/fatty acid metabolism**										
162.112	13.3	* Carnitine	<0.001	0.253	<0.001	0.164	ns	1.065	<0.001	0.676
204.123	11.0	* Acetylcarnitine	<0.001	0.273	<0.001	0.050	ns	1.016	<0.001	0.190
232.154	8.7	Butanoylcarnitine	<0.001	14.083	ns	1.992	ns	0.903	<0.001	0.131
664.117	14.3	* NAD+	<0.001	0.487	<0.001	0.135	<0.001	1.228	<0.001	0.336
**Miscellaneous**										
104.106	19.6	* Choline	<0.001	0.019	<0.001	0.231	<0.001	1.423	<0.001	7.270
166.086	10.0	* Phenylalanine	<0.001	0.381	<0.05	0.873	<0.01	1.157	<0.001	2.358
118.086	12.4	* Valine	<0.001	1.148	<0.01	2.257	<0.01	1.148	<0.001	2.257
120.065	14.3	* Threonine	<0.001	0.610	ns	0.948	<0.05	1.140	<0.001	1.704
88.040	14.7	* Alanine	ns	0.965	<0.001	0.385	ns	1.098	<0.001	0.442
179.056	17.1	* Hexose	<0.001	0.564	<0.001	0.364	<0.01	0.931	<0.001	0.605
195.051	13.7	* Gluconic acid	<0.001	0.559	<0.001	0.103	<0.001	0.837	<0.001	0.154
258.110	14.4	* Glycerophosphocholine	<0.001	0.020	<0.001	0.031	<0.001	1.529	<0.001	2.342

RT: retention time (in min); MR: melittin treated A2780CR cells; R: untreated A2780CR cells; MS: melittin treated A2780 cells; S: untreated A2780 cells; ns: non-significant. * Retention time matches standard on ZIC-pHILIC column; ** Retention time matches standard on ZIC-HILIC column.

## Supplementary Materials

The following are available online at  http://www.mdpi.com/2218-1989/6/4/35/s1, Table S1: Differences in the top 50 lipids between A2780 cells and A2780CR cells before and after melittin treatment, Figure S1: Cell viability determined following treatment with cisplatin for 24 h (A) IC_50_ = 10.8 µg/mL A2780CR; (B) IC_50_ = 4.9 µg/mL A2780. Figure S2: Layout of carbon sources in the wells on the PM-M1 microplate, Figure S3: Changes in the metabolism of ovarian cancer A2780 and A2780CR cells, Figure S4: Lactate dehydrogenase (LDH) assay, Figure S5: Effect of Melittin on caspase-3 activity in A2780 and A2780CR cells.

## References

[1] Vermeersch K.A., Styczynski M.P. (2013). Applications of metabolomics in cancer research. J. Carcinog..

[2] Vander Heiden M.G., Cantley L.C., Thompson C.B. (2009). Understanding the Warburg effect: The metabolic requirements of cell proliferation. Science.

[3] Poisson L.M., Munkarah A., Madi H., Datta I., Hensley-Alford S., Tebbe C., Buekers T., Giri S., Rattan R. (2015). A metabolomic approach to identifying platinum resistance in ovarian cancer. J. Ovarian Res..

[4] Wang D., Lippard S.J. (2005). Cellular processing of platinum anticancer drugs. Nat. Rev. Drug Discov..

[5] Matsuo K., Eno M.L., Im D.D., Rosenshein N.B., Sood A.K. (2010). Clinical relevance of extent of extreme drug resistance in epithelial ovarian carcinoma. Gynecol. Oncol..

[6] Zwelling L.A., Kohn K.W. (1978). Mechanism of action of cis-dichlorodiammineplatinum (II). Cancer Treat. Rep..

[7] Siddik Z.H. (2003). Cisplatin: Mode of cytotoxic action and molecular basis of resistance. Oncogene.

[8] Galluzzi L., Senovilla L., Vitale I., Michels J., Martins I., Kepp O., Castedo M., Kroemer G. (2012). Molecular mechanisms of cisplatin resistance. Oncogene.

[9] Mandic A., Hansson J., Linder S., Shoshan M.C. (2003). Cisplatin induces endoplasmic reticulum stress and nucleus-independent apoptotic signaling. J. Biol. Chem..

[10] Rabik C.A., Dolan M.E. (2007). Molecular mechanisms of resistance and toxicity associated with platinating agents. Cancer Treat. Rev..

[11] Byun S.S., Kim S.W., Choi H., Lee C., Lee E. (2005). Augmentation of cisplatin sensitivity in cisplatin-resistant human bladder cancer cells by modulating glutathione concentrations and glutathione-related enzyme activities. BJU Int..

[12] Jeong Y.-J., Choi Y., Shin J.-M., Cho H.-J., Kang J.-H., Park K.-K., Choe J.-Y., Bae Y.-S., Han S.-M., Kim C.-H. (2014). Melittin suppresses EGF-induced cell motility and invasion by inhibiting PI3K/Akt/mTOR signaling pathway in breast cancer cells. Food Chem. Toxicol..

[13] Kohno M., Horibe T., Ohara K., Ito S., Kawakami K. (2014). The Membrane-Lytic Peptides K8L9 and Melittin Enter Cancer Cells via Receptor Endocytosis following Subcytotoxic Exposure. Chem. Biol..

[14] Mahmoodzadeh A., Morady A., Zarrinnahad H., Ghasemi-Dehkordi P., Mahdavi M., Shahbazzadeh D., Shahmorady H. (2013). Isolation of melittin from bee venom and evaluation of its effect on proliferation of gastric cancer cells. Tehran Univ. Med. Sci..

[15] Mahmoodzadeh A., Zarrinnahad H., Bagheri K.P., Moradia A., Shahbazzadeh D. (2015). First report on the isolation of melittin from Iranian honey bee venom and evaluation of its toxicity on gastric cancer AGS cells. J. Chin. Med. Assoc..

[16] Jo M., Park M.H., Kollipara P.S., An B.J., Song H.S., Han S.B., Kim J.H., Song M.J., Hong J.T. (2012). Anti-cancer effect of bee venom toxin and melittin in ovarian cancer cells through induction of death receptors and inhibition of JAK2/STAT3 pathway. Toxicol. Appl. Pharmacol..

[17] Liu M., Zong J., Liu Z., Li L., Zheng X., Wang B., Sun G. (2013). A novel melittin-MhIL-2 fusion protein inhibits the growth of human ovarian cancer SKOV3 cells in vitro and in vivo tumor growth. Cancer Immunol. Immunother..

[18] Qian C.-Y., Wang K.-L., Fang F.-F., Gu W., Huang F., Wang F.-Z., Li B., Wang L.-N. (2015). Triple-controlled oncolytic adenovirus expressing melittin to exert inhibitory efficacy on hepatocellular carcinoma. Int. J. Clin. Exp. Pathol..

[19] Wu X., Zhao B., Cheng Y., Yang Y., Huang C., Meng X., Wu B., Zhang L., Lv X., Li J. (2015). Melittin induces PTCH1 expression by down-regulating MeCP2 in human hepatocellular carcinoma SMMC-7721 cells. Toxicol. Appl. Pharmacol..

[20] Oh S.-B., Hwang C.J., Song S.-Y., Jung Y.Y., Yun H.-M., Sok C.H., Sung H.C., Yi J.-M., Park D.H., Ham Y.W. (2014). Anti-cancer effect of tectochrysin in NSCLC cells through overexpression of death receptor and inactivation of STAT3. Cancer Lett..

[21] Gajski G., Garaj-Vrhovac V. (2013). Melittin: A lytic peptide with anticancer properties. Environ. Toxicol. Pharmacol..

[22] Shin J.-M., Jeong Y.-J., Cho H.-J., Park K.-K., Chung I.-K., Lee I.-K., Kwak J.-Y., Chang H.-W., Kim C.-H., Moon S.-K. (2013). Melittin suppresses HIF-1α/VEGF expression through inhibition of ERK and mTOR/p70S6K pathway in human cervical carcinoma cells. PLoS ONE.

[23] Zhang T., Watson D.G., Wang L., Abbas M., Murdoch L., Bashford L., Ahmad I., Lam N.Y., Ng A.C., Leung H.Y. (2013). Application of Holistic Liquid Chromatography-High Resolution Mass Spectrometry Based Urinary Metabolomics for Prostate Cancer Detection and Biomarker Discovery. PLoS ONE.

[24] Zhang T., Watson D.G. (2015). A short review of applications of liquid chromatography mass spectrometry based metabolomics techniques to the analysis of human urine. Analyst.

[25] Zhang R., Zhang T., Ali A.M., Al Washih M., Pickard B., Watson D.G. (2016). Metabolomic Profiling of Post-Mortem Brain Reveals Changes in Amino Acid and Glucose Metabolism in Mental Illness Compared with Controls. Comput. Struct. Biotechnol. J..

[26] Frezza C., Zheng L., Tennant D.A., Papkovsky D.B., Hedley B.A., Kalna G., Watson D.G., Gottlieb E. (2011). Metabolic profiling of hypoxic cells revealed a catabolic signature required for cell survival. PLoS ONE.

[27] Beger R.D. (2013). A review of applications of metabolomics in cancer. Metabolites.

[28] Griffin J.L., Shockcor J.P. (2004). Metabolic profiles of cancer cells. Nat. Rev. Cancer.

[29] Čuperlović-Culf M., Barnett D.A., Culf A.S., Chute I. (2010). Cell culture metabolomics: Applications and future directions. Drug Discov. Today.

[30] Bochner B.R., Siri M., Huang R.H., Noble S., Lei X.H., Clemons P.A., Wagner B.K. (2011). Assay of the multiple energy-producing pathways of mammalian cells. PLoS ONE.

[31] Vermeersch K.A., Wang L., McDonald J.F., Styczynski M.P. (2014). Distinct metabolic responses of an ovarian cancer stem cell line. BMC Syst. Biol..

[32] Wang J., Jin L., Li X., Deng H., Chen Y., Lian Q., Ge R., Deng H. (2013). Gossypol induces apoptosis in ovarian cancer cells through oxidative stress. Mol. BioSyst..

[33] Sasada S., Miyata Y., Tsutani Y., Tsuyama N., Masujima T., Hihara J., Okada M. (2013). Metabolomic analysis of dynamic response and drug resistance of gastric cancer cells to 5-fluorouracil. Oncol. Rep..

[34] Schuber F. (1989). Influence of polyamines on membrane functions. Biochem. J..

[35] Moinard C., Cynober L., de Bandt J.-P. (2005). Polyamines: Metabolism and implications in human diseases. Clin.Nutr..

[36] Morré D.J., Kartenbeck J., Franke W.W. (1979). Membrane flow and interconversions among endomembranes. Biochim. Biophys. Acta Rev. Biomembr..

[37] Seiler N., Raul F. (2005). Polyamines and apoptosis. J. Cell. Mol. Med..

[38] Uetaki M., Tabata S., Nakasuka F., Soga T., Tomita M. (2015). Metabolomic alterations in human cancer cells by vitamin C-induced oxidative stress. Sci. Rep..

[39] Moore Z., Boothman D.A. (2014). Tumor-specific targeting of the NAD metabolome with β-lapachone and NamPT inhibition. Cancer Res..

[40] Komatsu N., Nakagawa M., Oda T., Muramatsu T. (2000). Depletion of Intracellular NAD+ and ATP Levels during Ricin-Induced Apoptosis through the Specific Ribosomal Inactivation Results in the Cytolysis of U937 Cells. J. Biochem..

[41] Tolstikov V., Nikolayev A., Dong S., Zhao G., Kuo M.-S. (2014). Metabolomics Analysis of Metabolic Effects of Nicotinamide Phosphoribosyltransferase (NAMPT) Inhibition on Human Cancer Cells. PLoS ONE.

[42] Ben Sellem D., Elbayed K., Neuville A., Moussallieh F.M., Lang-Averous G., Piotto M., Bellocq J.P., Namer I.J. (2011). Metabolomic Characterization of Ovarian Epithelial Carcinomas by HRMAS-NMR Spectroscopy. J. Oncol..

[43] Denkert C., Budczies J., Kind T., Weichert W., Tablack P., Sehouli J., Niesporek S., Könsgen D., Dietel M., Fiehn O. (2006). Mass spectrometry–based metabolic profiling reveals different metabolite patterns in invasive ovarian carcinomas and ovarian borderline tumors. Cancer Res..

[44] Halama A., Guerrouahen B.S., Pasquier J., Diboun I., Karoly E.D., Suhre K., Rafii A. (2015). Metabolic signatures differentiate ovarian from colon cancer cell lines. J. Transl. Med..

[45] Zammit V.A., Ramsay R.R., Bonomini M., Arduini A. (2009). Carnitine, mitochondrial function and therapy. Adv. Drug Deliv. Rev..

[46] Veldman R.J., Klappe K., Hinrichs J., Hummel I., van der Schaaf G., Sietsma H., Kok J.W. (2002). Altered sphingolipid metabolism in multidrug-resistant ovarian cancer cells is due to uncoupling of glycolipid biosynthesis in the Golgi apparatus. FASEB J..

[47] Lavie Y., Cao H.-T., Bursten S.L., Giuliano A.E., Cabot M.C. (1996). Accumulation of Glucosylceramides in Multidrug-resistant Cancer Cells. J. Biol. Chem..

[48] May G.L., Wright L.C., Dyne M., Mackinnon W.B., Fox R.M., Mountford C.E. (1988). Plasma membrane lipid composition of vinblastine sensitive and resistant human leukaemic lymphoblasts. Int. J. Cancer.

[49] Yonezawa A., Inui K.-I. (2011). Organic cation transporter OCT/SLC22A and H+/organic cation antiporter MATE/SLC47A are key molecules for nephrotoxicity of platinum agents. Biochem. Pharmacol..

[50] Tusiimire J., Wallace J., Dufton M., Parkinson J., Clements C.J., Young L., Park J.K., Jeon J.W., Watson D.G. (2015). An LCMS method for the assay of melittin in cosmetic formulations containing bee venom. Anal. Bioanal. Chem..

[51] Zhang R., Watson D.G., Wang L., Westrop G.D., Coombs G.H., Zhang T. (2014). Evaluation of mobile phase characteristics on three zwitterionic columns in hydrophilic interaction liquid chromatography mode for liquid chromatography-high resolution mass spectrometry based untargeted metabolite profiling of Leishmania parasites. J. Chromatogr. A.

[52] Zheng L., T’Kind R., Decuypere S., von Freyend S.J., Coombs G.H., Watson D.G. (2010). Profiling of lipids in Leishmania donovani using hydrophilic interaction chromatography in combination with Fourier transform mass spectrometry. Rapid Commun. Mass Spectrom..

[53] Pluskal T., Castillo S., Villar-Briones A., Oresic M. (2010). MZmine 2: Modular framework for processing, visualizing, and analyzing mass spectrometry-based molecular profile data. BMC Bioinform..

[54] Katajamaa M., Orešič M. (2005). Processing methods for differential analysis of LC/MS profile data. BMC Bioinform..

[55] Carrasco R.A., Stamm N.B., Patel B.K. (2003). One-step cellular caspase-3/7 assay. Biotechniques.

