# Supplementary material for: Metabolomic Profiling of the Effects of Melittin on Cisplatin Resistant and Cisplatin Sensitive Ovarian Cancer Cells Using Mass Spectrometry and Biolog Microarray Technology
Source: Metabolites. 2016 Oct 13;6(4):35. doi: 10.3390/metabo6040035 (PMC5192441; doi:10.3390/metabo6040035)
Supplement: Supplementary file 1 [file metabolites-06-00035-s001.pdf]

Supplementary Materials: Metabolomic Profiling of the Effects of Melittin on Cisplatin Resistant and Cisplatin Sensitive Ovarian Cancer Cells Using Mass Spectrometry and Biolog Microarray Technology

Sanad Alonezi, Jonans Tusiimire, Jennifer Wallace, Mark J. Dufton, John A. Parkinson, Louise C. Young, Carol J. Clements, Jin Kyu Park, Jong Woon Jeon, Valerie A. Ferro and David G. Watson

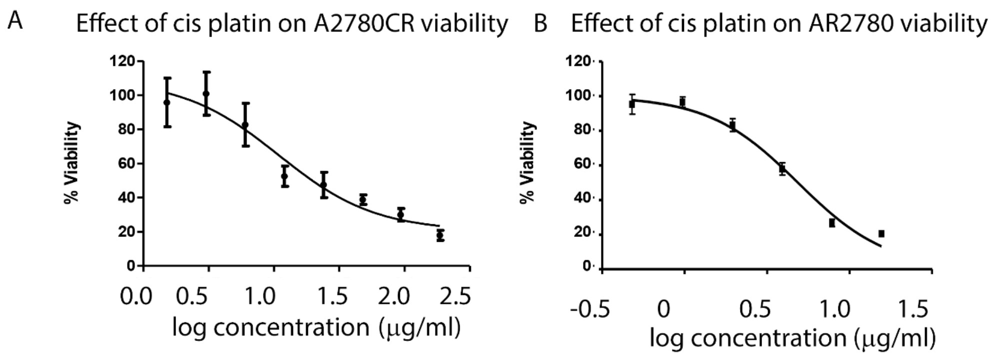

Figure S1. Cell viability was determined following treatment with cisplatin for 24 h (**A**) IC_{50} = 10.8 µg/mL A2780CR; (**B**) IC_{50} = 4.9 µg/mL A2780.

PM-M1 MicroPlate™ - Carbon and Energy Sources

A1 Negative Control	A2 Negative Control	A3 Negative Control	A4 α-Cyclodextrin	A5 Dextrin	A6 Glycogen	A7 Maltitol	A8 Maltotriose	A9 D-Maltose	A10 D-Trehalose	A11 D-Cellobiose	A12 β-Gentiobiose
B1 D-Glucose-6-Phosphate	B2 α-D-Glucose-1-Phosphate	B3 L-Glucose	B4 α-D-Glucose	B5 α-D-Glucose	B6 α-D-Glucose	B7 3-O-Methyl-D-Glucose	B8 α-Methyl-D-Glucoside	B9 β-Methyl-D-Glucoside	B10 D-Salicin	B11 D-Sorbitol	B12 N-Acetyl-D-Glucosamine
C1 D-Glucosaminic Acid	C2 D-Glucuronic Acid	C3 Chondroitin-6-Sulfate	C4 Mannan	C5 D-Mannose	C6 α-Methyl-D-Mannoside	C7 D-Mannitol	C8 N-Acetyl-β-D-Mannosamine	C9 D-Melezitose	C10 Sucrose	C11 Palatinose	C12 D-Turanose
D1 D-Tagatose	D2 L-Sorbose	D3 L-Rhamnose	D4 L-Fucose	D5 D-Fucose	D6 D-Fructose-6-Phosphate	D7 D-Fructose	D8 Stachyose	D9 D-Raffinose	D10 D-Lactitol	D11 Lactulose	D12 α-D-Lactose
E1 Melibionic Acid	E2 D-Melibiose	E3 D-Galactose	E4 α-Methyl-D-Galactoside	E5 β-Methyl-D-Galactoside	E6 N-Acetyl-Neuraminic Acid	E7 Pectin	E8 Sedoheptulosan	E9 Thymidine	E10 Uridine	E11 Adenosine	E12 Inosine
F1 Adonitol	F2 L- Arabinose	F3 D-Arabinose	F4 β-Methyl-D-Xylopyranoside	F5 Xylitol	F6 Myo-Inositol	F7 Meso-Erythritol	F8 Propylene glycol	F9 Ethanolamine	F10 D,L- α-Glycerol-Phosphate	F11 Glycerol	F12 Citric Acid
G1 Tricarballylic Acid	G2 D,L-Lactic Acid	G3 Methyl D-lactate	G4 Methyl pyruvate	G5 Pyruvic Acid	G6 α-Keto-Glutaric Acid	G7 Succinamic Acid	G8 Succinic Acid	G9 Mono-Methyl Succinate	G10 L-Malic Acid	G11 D-Malic Acid	G12 Meso-Tartaric Acid
H1 Acetoacetic Acid (a)	H2 γ-Amino-N-Butyric Acid	H3 α-Keto-Butyric Acid	H4 α-Hydroxy-Butyric Acid	H5 D,L-β-Hydroxy-Butyric Acid	H6 γ-Hydroxy-Butyric Acid	H7 Butyric Acid	H8 2,3-Butanediol	H9 3-Hydroxy-2-Butanone	H10 Propionic Acid	H11 Acetic Acid	H12 Hexanoic Acid

Figure S2. Layout of carbon sources in the wells on the PM-M1 microplate.

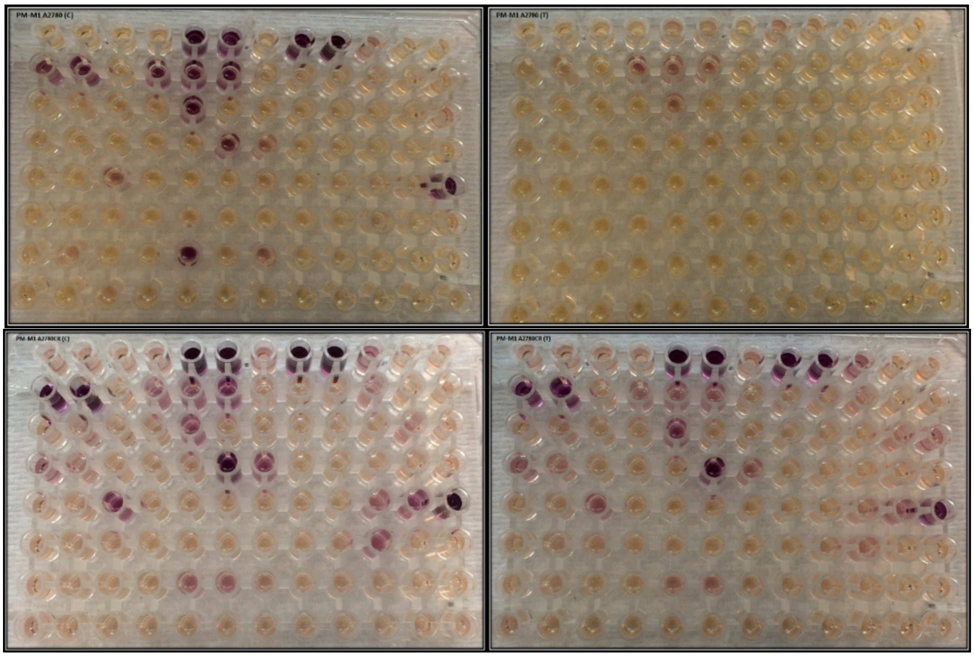

Figure S3. Changes in the metabolism of ovarian cancer A2780 and A2780CR cells. Top left was A2780 without treated. Top right was A2780 after exposure to melittin. Bottom left was A2780CR without treated. Bottom right was A2780 after exposure to melittin.

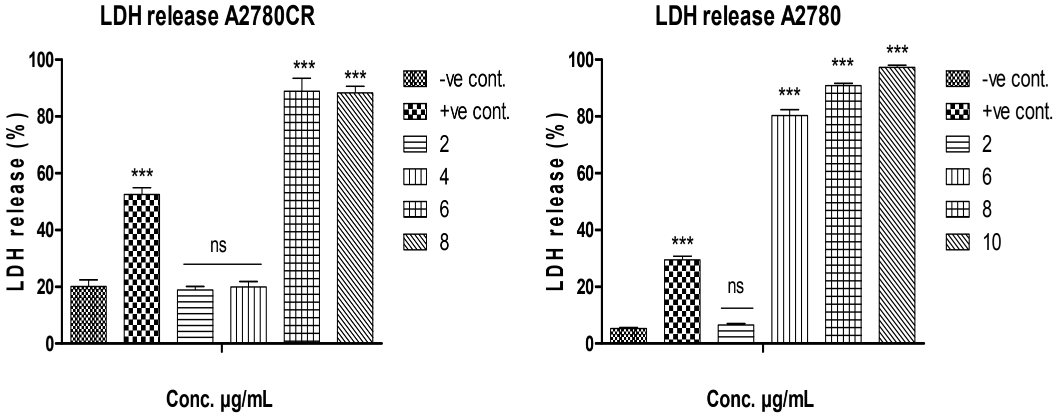

Figure S4. Lactate dehydrogenase (LDH) assay. Effect of melittin on leakage of lactate dehydrogenase (LDH) from A2780 and A2780CR cell lines. The cells were incubated with melittin at different concentrations for 24 h. LDH activity was measured at 490 nm using an LDH cytotoxicity kit. Data were expressed as the mean ± SD of three independent experiments. Significant difference in LDH activity of melittin compared to untreated cells was tested by one-way ANOVA followed by Bonferroni's Multiple Comparison test to determine the differences between the experimental groups. Differences were considered significant at $p < 0.001$ (∗∗∗) and ns: no significance.

% Cytotoxicity = Experimental − Culture Medium Background × 100

Maximum LDH Release − Culture Medium Background

Effect of Melittin on Caspase-3 Activity

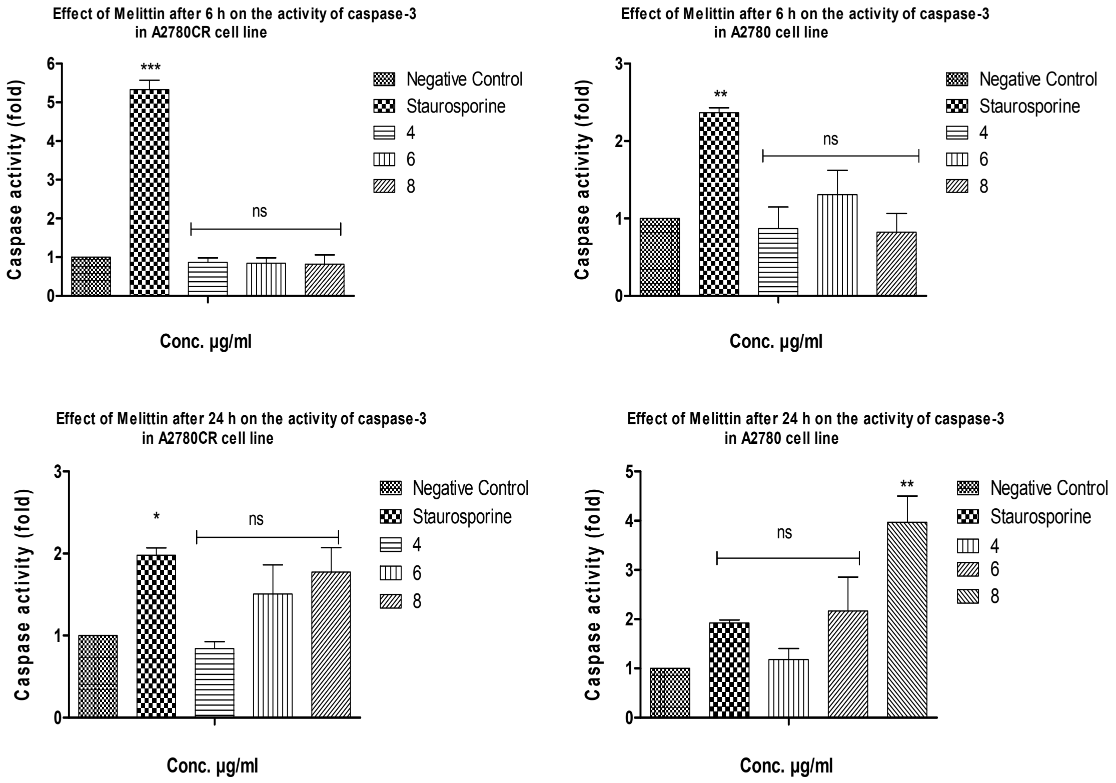

Figure S5. Effect of Melittin on caspase-3 activity in A2780 and A2780CR cells. Both cell lines were incubated with different concentrations of melittin to measure caspase-3 activity. Staurosporine (10 µM) was used as a positive control. Following 6 and 24 h, cell were incubated with the caspase detection buffer and the fluorescence signal was measured following 1 h at 360 nm (excitation) and at 460 nm (emission). Data are presented as the fold change compare to untreated cells (negative control). Data were expressed as the mean ± SD of three independent experiments. Significant difference in caspase-3 activity of melittin compared to untreated cells was tested by one-way ANOVA followed by Bonferroni's Multiple Comparison test to determine the differences between the experimental groups. Differences were considered significant at the level of $p < 0.05$ and ns: no significance.

Table S1. Differences in the top 50 lipids between A2780 cells and A2780CR cells before and after melittin treatment.

m/z	Rt(min)	Met Name	*p* Value S/R	Ratio S/R	MS/S *p* Value	Ratio MS/S	MR/R *p* Value	MR/R Ratio	MS/MR *p* Value	Ratio MS/MR
760.5846	13.9	PC34:1	0.03	1.36	0.01	0.64	0.09	0.82	0.58	1.05
786.6	13.9	PC36:2	0.29	1.10	0.03	0.75	0.12	0.85	0.77	0.97
732.5539	14.0	PC32:1	0.01	1.87	0.01	0.56	0.00	0.59	< 0.001	1.76
746.6055	14.1	PC34:0	< 0.001	3.28	0.03	0.74	0.07	0.82	< 0.001	2.95
788.6156	13.9	PC36:1	0.94	0.99	0.02	0.65	0.33	1.11	0.02	0.58
734.5691	14.0	PC32:0	0.01	1.44	0.01	0.61	0.04	0.79	0.31	1.10
758.5694	13.9	PC34:2	0.01	1.58	0.02	0.73	0.01	0.69	< 0.001	1.68
703.5745	14.5	SM14:1	< 0.001	1.87	0.63	0.96	0.69	1.05	< 0.001	1.72
720.5899	14.2	PC32:2 ether lipid	< 0.001	4.62	0.12	0.82	0.01	0.69	< 0.001	5.50
718.5746	14.1	PC32:0 ether lipid	0.00	3.31	0.11	0.83	0.17	1.17	< 0.001	2.35
706.5382	14.1	PC30:0	0.03	1.31	0.00	0.49	0.00	0.61	0.47	1.06
784.5847	13.8	PC36:3	0.02	1.58	0.05	0.76	0.45	0.93	0.03	1.30
782.5672	13.7	PC36:4	0.01	1.76	0.57	0.95	0.18	1.20	0.03	1.39
768.5529	9.7	PC38:4	0.01	1.87	0.23	1.12	0.00	1.41	< 0.001	1.48
808.5836	13.7	PC38:5	0.03	1.64	0.32	1.12	0.11	1.30	0.03	1.41
810.5995	13.7	PC38:4	0.02	1.88	0.44	0.90	0.02	1.84	0.54	0.93
768.5885	13.8	PC36:3 ether lipid	< 0.001	4.56	0.78	0.98	0.01	1.62	0.00	2.75
744.5905	13.9	PC34:1 ether lipid	< 0.001	1.88	0.45	0.94	0.17	1.19	0.01	1.49
794.6051	13.8	PC38:5 ether lipid	< 0.001	3.58	0.11	1.19	0.01	2.11	< 0.001	2.01
796.6206	13.8	PC38:4 ether lipid	< 0.001	5.72	0.39	0.92	0.01	1.73	< 0.001	3.03
752.5584	9.6	PE38:5	< 0.001	3.20	0.51	0.94	0.01	1.31	< 0.001	2.29
813.6838	14.4	SM42:2	< 0.001	1.84	0.88	0.99	0.62	1.07	< 0.001	1.71
804.5759	14.0	PS37:0	0.69	0.96	< 0.001	1.32	0.13	1.14	0.05	1.11
812.6155	13.8	PC38:3	0.01	1.84	0.01	0.60	0.30	1.14	0.80	0.97
814.6312	13.9	PC38:2	0.01	0.67	0.02	0.60	0.08	0.81	0.01	0.49
744.5534	10.1	PE36:2 ether lipid	0.26	0.89	0.02	0.69	0.01	0.75	0.06	0.82
772.621	14.0	PC 36:1 ether lipid	0.00	2.08	0.06	0.80	0.81	0.97	0.00	1.70
300.2893	10.4	Dehydrosphinganine	0.00	6.21	0.00	0.66	0.00	0.62	0.00	6.63
774.6007	13.9	PC35:1	0.08	1.24	0.13	0.84	0.13	1.21	0.21	0.87
752.5583	9.6	PE38:5 ether lipid	0.01	3.33	0.21	0.80	0.03	1.27	0.00	2.12
766.5367	9.6	PE38:5	0.03	1.99	0.35	0.85	0.44	0.95	0.00	1.80
750.5427	9.6	PE38:4 ether lipid	0.01	1.78	0.39	0.92	0.50	1.04	0.00	1.57
724.5273	9.7	PE36:5 ether lipid	0.02	1.51	0.74	1.03	0.01	1.41	0.14	1.11

772.5851	13.9	PC35:2	0.03	1.41	0.42	0.93	0.33	0.90	0.01	1.45
774.6365	14.1	PC36:0 ether lipid	0.00	3.40	0.02	0.69	0.03	0.76	0.00	3.11
770.6052	13.9	PC36:2 ether lipid	0.00	2.70	0.01	0.63	0.55	1.07	0.01	1.58
731.6057	14.5	SM36:2	0.00	2.97	0.24	1.10	0.63	1.07	0.00	3.07
718.538	10.3	PE34:1	0.16	1.14	0.00	0.58	0.00	0.61	0.42	1.08
692.5589	14.2	PC30:2 ether lipid	0.00	3.45	0.02	0.62	0.02	0.77	0.00	2.80
730.5382	13.9	PC32:2	0.00	3.79	0.01	0.70	0.00	0.58	0.00	4.61
862.6244	3.1	C18:0 Lactosylceramide	< 0.001	5.11	0.20	0.86	0.03	1.16	0.00	3.79
746.569	10.2	PE34:1 ether lipid	0.01	0.69	0.01	0.66	0.03	0.78	0.01	0.59
792.5897	13.7	PC38:6 ether lipid	0.01	2.36	0.81	1.03	0.02	1.91	0.08	1.27
282.2788	10.4	Octadecenamide	0.00	5.26	0.00	0.67	0.00	0.62	0.00	5.68
834.5994	13.7	PC40:6	0.68	1.05	0.08	0.74	0.07	1.42	0.02	0.55
836.6151	13.7	PC40:5	0.33	1.16	0.17	0.79	0.10	1.34	0.06	0.68
863.5661	3.7	PI36:1	0.07	0.87	0.09	1.15	0.08	1.22	0.07	0.82
790.5586	11.4	PS 36:1	0.28	1.10	0.03	0.74	0.12	0.87	0.37	0.93
756.5523	13.9	PC34:3	0.00	2.16	0.04	0.81	0.02	0.76	0.00	2.33
887.5643	3.6	PI38:3	0.00	2.32	0.00	1.52	0.58	0.96	0.00	3.66
